# Microservice architecture based self-service drone aerial photography system for smart cultural tourism: Design and implementation

**DOI:** 10.1371/journal.pone.0343832

**Published:** 2026-08-10

**Authors:** Zehua Liu, Xiaoyou Yu, Xiaobao Liu, Qian Hu

**Affiliations:** 1 Jusup Balasagyn Kyrgyz National University, Bishkek, Kyrgyz Republic; 2 Lab of Integrated Sensing, Communications, Computing and Control; Hunan University, Changsha, Hunan Province, China; 3 Shenzhen MSU-BIT University, Shenzhen, Guangdong Province, China; Xi'an Jiaotong-Liverpool University, CHINA

## Abstract

Although the low-altitude economy has been designated a strategic emerging industry, consumer-grade drone aerial photography in cultural tourism remains constrained by three coupled bottlenecks: dependence on licensed pilots, fragmented and manually orchestrated service workflows, and post-production cycles measured in hours to days. These bottlenecks jointly prevent drone aerial photography from being delivered as a standardized, on-demand consumer service. This study aims to design, implement, and empirically evaluate a software architecture that transforms professional drone aerial photography into an automated, scan-to-use consumer service, and to test whether a microservice-based design can simultaneously meet the latency, reliability, and cost requirements of mass-market smart tourism deployment. We propose KanYiKan, a distributed microservice system in which (i) an MQTT-based bidirectional channel connects the cloud scheduler with heterogeneous ground control stations, (ii) order lifecycles are governed by a deterministic finite-state machine with optimistic-locking transitions, (iii) drone assignment is formulated as a weighted multi-objective scoring problem balancing battery margin, spatial proximity, and historical reliability, and (iv) an automated video pipeline performs no-reference rule-based quality filtering, template-based semantic matching using a pre-trained vision encoder, and rendering. The system is evaluated through functional verification, JMeter-based concurrent stress testing (100–1000 users), fault-injection robustness testing, and a comparative analysis against the traditional pilot-mediated workflow. Under simulated high-concurrency loads, P99 latency of core APIs stayed at or below 312 ms and error rates below 0.05%; per-service cost dropped by over 90% (from 500–3000 CNY to 50–100 CNY), end-to-end delivery shrank from 24–72 hours to 15–30 minutes, and the pilot-per-order requirement was eliminated. All five injected fault scenarios were handled without service-wide outage. The results provide empirical evidence that a cloud-native microservice architecture, combined with FSM-driven workflow control and lightweight multi-objective scheduling, is a viable software paradigm for democratizing UAV services and offers a replicable reference design for scalable low-altitude applications in smart cultural tourism.

## Introduction

In the last 20 years, software architecture has undergone a paradigm shift from monolithic architecture to service-oriented architecture and, more recently, to microservices [1,2]. Microservices break down complex applications into small, independently deployable services that can be used to perform a single business capability [3]. This architectural style has many benefits such as loose coupling, high cohesion, independent scaling and technology heterogeneity [4]. The use of cloud-native technologies such as containerization using Docker, and orchestration using Kubernetes, has also accelerated the adoption of microservices [5]. These progressions have empowered organizations to adopt microservices as the prevailing model for building and launching modern distributed systems that are quick to develop, simple to fault isolate and simple to elastically scale [6].

Microservices architecture has been embraced in web services and has recently been observed in Internet of Things (IoT) and robotic systems [7,8]. Like microservices, IoT systems operate in a distributed and event-driven manner, with streams of data always coming from a vast sea of sorts of devices and that data always needs to be processed in real-time [9]. In the world of robotics, ROS 2 has taken a microservices-oriented approach with increased distributed deployment and real-time communication capabilities [10,11]. Publish-subscribe communication patterns are increasingly important in resource-constrained environments, where message-oriented communication protocols such as MQTT are in the spotlight [12]. As these technological developments have been made, there are only a few practical papers in the literature to date addressing the use of microservices architecture in the context of UAV service systems, mostly related to enterprise-level fleet management systems, but not to the service platform directly facing end-users [13].

Most of the currently available UAV software packages are built on top of existing software, like PX4 or ArduPilot and are based on MAVLink protocol, which is a communication protocol between the flight controller and ground control station [14,15]. These systems are great for flight control and mission execution, but are primarily conceived at the lower level for operations of vehicles and not for higher-level business service integration [16]. Most of the current UAV management systems are monolithic and tightly coupled, and therefore do not exhibit good characteristics of extensibility and scalability [17]. Supporting high concurrency of users and dynamic allocation of resources becomes challenging, as well as seamless integration with third-party services such as payment gateways and AI processing pipelines [18]. Further, the majority of current solutions are geared towards professional users and not towards the general consumer, which makes the user experience hard to navigate and the learning curve steep, thus hindering mass-market adoption [19]. Lack of consumer-friendly, lightweight interaction paradigms is a key missing element in relation to the democratization of UAV-based services [20].

In other engineering disciplines, the ability to break down tight pipelines of information into modular, service-oriented components is a pre-condition for scalability, reusability and downstream automation, and it is something that has been proven more than once. Similar architectural decoupling has been reported in process-engineering areas, where complex multi-stage processes need to be made reproducible and adaptive, such as multi-stage treatment and recycling pipelines for emerging contaminants [21–23], optimization of multi-objective material recovery processes with explicit performance trade-offs [24], and data-driven pipelines that involve physical sensing and computational decision layers [25]. While the works are not specifically for UAV services, they all have one goal in common: that when a workflow ties together resources of different types, and resources are tied to end-user demand, a modular plus quantitative scheduling approach can be more effective than monolithic approaches that are manually orchestrated. This is what motivates the present work, as there is no analogous modular design of a UAV aerial photography system for consumers in the context of smart tourism that is backed with evidence.

Smart tourism is a paradigm shift in the tourism sector which uses information and communication technologies to improve the tourist experience, making it more personalized, real-time and immersive [26,27]. Such a domain demands a high level of availability, real-time response and user-friendly interfaces with no technical jargon, usable by non-technical users [28,29]. One of the most interesting smart tourism applications is aerial photography using UAVs, which is a technique that provides unique aerial views which can increase the tourists’ experience [30,31]. Smart tourism services involve lots of interactions with the users, payment processing, mission scheduling, flight control and the use of AI to edit the videos; this makes it a perfect testbed for testing microservices-based UAV system architectures [32,33]. If these various requirements could be solved, the practical feasibility of the architecture proposed would be proved.

The technology has been demonstrated, but there are still many gaps in the research between microservices architecture and consumer-oriented UAV services. The use of microservices in UAV service systems has not been extensively studied and systematic design patterns and best practices have not yet been identified. There is still limited literature on empirical assessments of microservices-based UAV platforms under realistic circumstances with metrics like number of concurrent requests handled, elastic scaling behaviour, and end-to-end latency. Solutions that tightly couple cloud-based scheduling with real-time drone communication using MQTT, AI-based video processing pipelines and lightweight mobile interfaces within a unified microservices framework are in particular missing.

Given the gaps above, the following guiding aim and objectives, which are testable, are offered for this study. The overall goal is to understand if a cloud-native microservice architecture will be able to bring the professional drone aerial photography to an automated consumer service while meeting latency, reliability and mass-market cost requirements of smart tourism. The specific objectives are to design a layered microservice reference architecture where user interaction, business logic, real-time communication with the drones and AI post-production are separated and can be deployed individually; to formalize the control of the order lifecycle as a deterministic finite-state machine that includes transaction-safe transitions to enforce that distributed order changes are consistent under concurrency and partial failure; to develop an algorithm for real-time scheduling of heterogeneous drones that simultaneously optimizes response latency, resource utilization and service reliability without sacrificing computational tractability for online decision-making; and to empirically evaluate the resulting system in terms of performance, robustness and efficiency criteria, and to compare it with the traditional pilot-mediated workflow on cost and delivery time. The implementation and evaluation objectives associated with these are detailed in the relevant implementation and evaluation sections of this paper.

To fill these gaps, a microservices-based fully automated self-service UAV aerial photography system, KanYiKan, is designed and implemented. The system consists of a Spring Boot-based microservices backend, an order scheduling engine based on a finite state machine, real-time communication with ground control stations using MQTT, an AI video processing pipeline, as well as a WeChat mini-program frontend. The system was deployed in an operational scenic area and was comprehensively tested for its functionality, high-concurrency performance, fault-injection robustness, and compared with the traditional pilot-mediated workflow in terms of efficiency. The Limitations section explicitly states a formal user-satisfaction study with validated instruments as a future work. Our contributions include a reference microservices architecture for consumer-facing UAV service platforms, empirical evidence demonstrating the technical feasibility and performance characteristics of such systems, and practical insights for the large-scale, standardized deployment of low-altitude economy applications in smart tourism contexts.

## System requirements analysis and overall architecture design

### User role definition

The system shall provide functionality for three basic roles of users: functional requirements shall be defined for these roles using use case analysis.

The tourists are C-end users and are the main recipients of services. They have basic needs such as browsing and searching scenic spots and packages through the WeChat mini-program, placing orders and making online payments through QR code scanning (with WeChat Pay support), and scheduling or instant triggering of aerial photography services, viewing real-time drone flight footage, push notifications on task status, and viewing, downloading, and sharing aerial photography products generated by AI.

The scenic area operators are B-end users, who will take care of the operational management in their respective area. They need to create, edit, and manage the availability of packages and attraction information, register drone hangars and equipment and monitor status and set up maintenance, plan, test, and publish preset flight routes, query order information and export statistical reports, and browse and manage the aerial footage library.

Platform-level operations and maintenance are the responsibility of system administrators. Their basic features are user permission management, user role management, setting system parameters, monitoring services, auditing logs, and handling exceptions and alerts.

It is an end-to-end service, that is, it is activated at the occurrence of a user or system event and has four different phases. During the ordering and payment phase, tourists choose the package, make an order, and the order is initially in unpaid state, after receiving the WeChat Pay call-back, the order is paid and can be scheduled. During the scheduling phase, paid orders are placed in a scheduling queue and then the scheduler assigns a real drone instance to each order based on drone availability, battery margin, and proximity. During the aerial execution phase, the drone used on the mission is launched, the flight path is flown and the drone finally lands, while flight telemetry and status updates are streamed back in real time. During the delivery phase, raw footage is uploaded, the post-production process is completed and the product is delivered to the user. In the implementation part, a formal state machine for the order lifecycle, with explicit transition rules and recovery paths, will be introduced later. This is a plain language description that contains enough information to describe the business logic at the requirements level.

### Overall system architecture design

#### Architecture design principles.

The system architecture design is based on four main principles.

High cohesion and low coupling means each microservice should focus on only one business domain and have loosely coupled services with well-defined API boundaries.

Elastic scalability means that the system can be scaled horizontally to handle increased traffic during holidays, based on business load.

Mechanisms like circuit breakers, degradation, and retries are needed for fault tolerance and self-healing, and hence the availability of the system.

Core business processes are implemented asynchronously using message queues to improve system throughput.

#### Layered architecture design.

**Fig 1:**
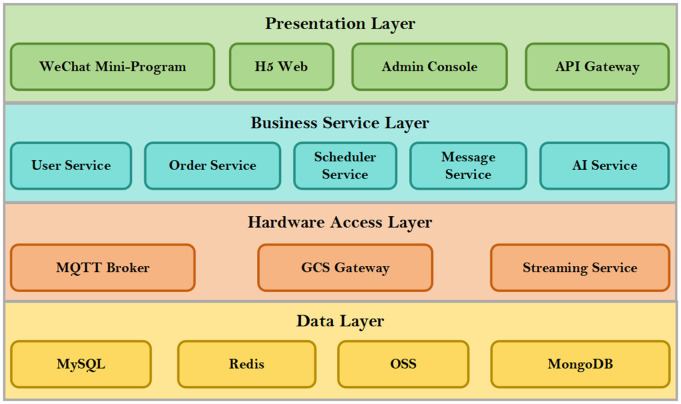
A diagram of a distributed microservice architecture with four layers.

The system is of a classic four-layer distributed microservice architecture, as shown in Fig 1.

There are a number of components in the Presentation Layer. The WeChat Mini-Program, based on the Uni-app framework, is the C-end user access point that can be deployed across platforms. The Backend Management System is built on Vue.js and Element UI as the B-end management console. The API Gateway, built with Spring Cloud Gateway, is for unified request routing, authentication, and rate limiting.

The Business Service Layer is a microservice cluster developed using Spring Boot 2.x, with the responsibilities of each service detailed in Table 1.

**Table 1:** Microservice responsibility definitions.

Service Name	Core Responsibilities	Key Dependencies
user-service	User registration, authentication, profile management	MySQL, Redis
order-service	Order CRUD, order state machine, payment interface	MySQL, Redis, MQ
scheduler-service	Scheduling and dispatching of resources, communication with GCS	Redis, MQTT
message-service	System notifications, template message push	Redis, WeChat API
file-service	Media file storage, CDN distribution	OSS, MySQL
ai-service	Video quality assessment, intelligent editing, synthesis	GPU, TensorFlow

There are a number of important components in the Hardware Access Layer. The EMQ X-based MQTT Broker guarantees reliable transmission at QoS levels 1 and 2. The GCS Gateway is a ground control station communication gateway, which parses and converts the MAVLink protocol. Real-time video stream forwarding relies on the Streaming Service, which is based on SRS.

The Data Layer comprises different storage options. MySQL 8.0 is used to store structured business data and high availability is achieved through master-slave replication. Redis 6.x offers distributed caching and distributed lock services to provide hot data access and atomicity of the scheduling. Drone telemetry data is semi-structured and stored with MongoDB. Object Storage Service is utilized for storing large quantities of raw aerial footage and finished products.

#### Technology selection rationale.

The various layers of the design were chosen specifically for their functionality and not because they were familiar or had been used before. We provide a summary of the key decisions and alternatives considered to make them more comprehensible so that practitioners can revisit them in the context of the deployment scenario they are considering.

The business service layer was decided on Spring Boot, as these services (order, payment, scheduling) require full-featured transactional support, extensive integration options (WeChat Pay, OSS, MQ), and long-running JVM behaviour under constant load. Node.js was also mentioned, as it uses less memory, but it was not appropriate for transactional workflows that span across multiple data stores; Go was interesting in terms of raw throughput, but did not integrate with the WeChat ecosystem as well at the time of design.

Redis is being used to cache hot data and as a distributed-lock provider in the scheduling. Unlike ZooKeeper and etcd, our scheduling workload has short-lived locks (sub-second), contention is moderate, and Redis is already required for caching. With Redis serving both purposes, no additional operational dependency was introduced, while still providing the atomicity of compare-and-swap state transitions described later.

The cloud-to-GCS link uses MQTT through EMQ X. MAVLink-over-TCP would have coupled the cloud directly to flight-control internals and offered no native publish/subscribe semantics for one-to-many command dispatch. gRPC streaming was investigated and determined not to be suitable, as it requires stable long-lived TCP connections that are not available on the mobile networks generally available at scenic locations. MQTT with QoS 1/2 allows specifying the level of reliability for each type of message and is able to cope with sporadic connectivity, as is the case in cultural tourism deployments.

Structured business state requires ACID semantics and is stored in MySQL. To handle high-volume, semi-structured telemetry without write amplification or schema rigidity, it is stored in MongoDB. Large binary footage is offloaded to OSS so that database storage is not consumed by media files and distribution through CDN is straightforward. Although a single-store design was considered, it was dismissed due to cost and operational considerations at the target scale.

These are not the only design decisions that can be made; each is driven by a concrete consumer drone-service workload requirement, and each can be revisited independently because the microservice boundaries isolate the impact of substitution.

### Data model design

The basic data entities and their relationships in the system may be represented as:


$$\begin{array}{c} ℳ=\langle ℰ,ℛ,𝒜\rangle \end{array}$$ (1)

where ℰ is the entity set, ℛ is the relationship set, and 𝒜 is the attribute mapping. The basic entities are defined as follows.

The User entity is defined as:


$$\begin{array}{c} User=\{id,openid,nickname,avatar,phone,create\_time\} \end{array}$$ (2)

The Order entity is defined as:


$$\begin{array}{c} Order=\{id,user\_id,combo\_id,drone\_id,status,amount,create\_time,...\} \end{array}$$ (3)

The Drone entity is defined as:


$$\begin{array}{c} Drone=\{id,sn,model,battery,status,location,hangar\_id,...\} \end{array}$$ (4)

The ComboMeal entity is defined as:


$$\begin{array}{c} ComboMeal=\{id,name,price,duration,route\_id,scenic\_id,...\} \end{array}$$ (5)

The relationships between entities can be expressed as:


$$\begin{array}{cc} R_{user\_order} & :User\overset{1:N\;}{\to }Order\\ R_{order\_drone} & :Order\overset{N:1\;}{\to }Drone\\ R_{combo\_route} & :ComboMeal\overset{N:1\;}{\to }Route \end{array}$$ (6)

## Scientific implementation of key technical modules

### FSM-based order lifecycle management

#### Formal definition of the state machine.

As the core business entity of the system, order lifecycle management is modeled using a Deterministic Finite Automaton. We adopt a formal DFA model here, rather than the plain-language description used in the requirements section, because the implementation must guarantee atomicity and consistency of state transitions under concurrent updates; without an explicit transition function it is not possible to specify which event sequences are legal and which must be rejected. The formal definition is as follows.

The order state machine ${𝒜}_{order}$ is defined as a quintuple:


$$\begin{array}{c} {𝒜}_{order}=(Q,\Sigma ,\delta ,q_{0},F) \end{array}$$ (7)

where *Q* represents the set of states containing $q_{unpaid}$, $q_{paid}$, $q_{queued}$, $q_{flying}$, $q_{landed}$, $q_{processing}$, $q_{done}$, $q_{cancelled}$, $q_{refunding}$, $q_{refunded}$, and $q_{error}$. The symbol Σ denotes the event alphabet including $e_{pay}$, $e_{cancel}$, $e_{timeout}$, $e_{dispatch}$, $e_{takeoff}$, $e_{complete}$, $e_{upload}$, $e_{render}$, $e_{refund}$, and $e_{error}$. The function δ:Q×Σ→Q represents the state transition function. The initial state is $q_{0}=q_{unpaid}$, and the set of final states is $F=\{q_{done},q_{cancelled},q_{refunded}\}$.

#### State transition function.

The key rules of the state transition function δ are defined as follows:


$$\begin{array}{c} \delta (q,e)=\left\{ \begin{array}{cc} q_{paid} & \text{if }q=q_{unpaid}\wedge e=e_{pay}\\ q_{cancelled} & \text{if }q=q_{unpaid}\wedge (e=e_{cancel}\vee e=e_{timeout})\\ q_{queued} & \text{if }q=q_{paid}\wedge e=e_{dispatch}\\ q_{flying} & \text{if }q=q_{queued}\wedge e=e_{takeoff}\\ q_{landed} & \text{if }q=q_{flying}\wedge e=e_{complete}\\ q_{processing} & \text{if }q=q_{landed}\wedge e=e_{upload}\\ q_{done} & \text{if }q=q_{processing}\wedge e=e_{render}\\ q_{refunding} & \text{if }q\in \{q_{paid},q_{queued}\}\wedge e=e_{refund}\\ q_{error} & \text{if }e=e_{error}\\ q & \text{otherwise, representing invalid transition} \end{array}\right. \end{array}$$ (8)

**Fig 2:**
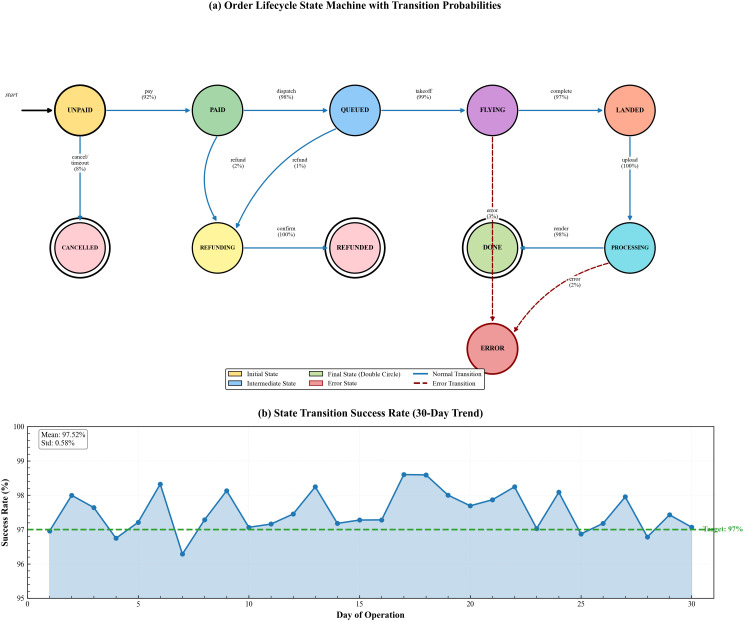
FSM-based order lifecycle management: **(a)** Order lifecycle state machine with transition probabilities showing all possible states and events; **(b)** State transition success rate over a 30-day operational period demonstrating system reliability above the 97% target threshold.

The complete state transition diagram is illustrated in Fig 2(a), and the operational reliability metrics are shown in Fig 2(b).

#### Transaction guarantees for state transitions.

To ensure atomicity and consistency of state transitions in a distributed environment, the system employs an optimistic locking mechanism based on Compare-And-Swap:


$$\begin{array}{c} \text{UPDATE}(order)=\left\{ \begin{array}{cc} \text{success} & \text{if }version_{db}=version_{expected}\\ \text{retry} & \text{if }version_{db}\neq version_{expected}\wedge retry<max\_retry\\ \text{fail} & \text{otherwise} \end{array}\right. \end{array}$$ (9)

The integrity constraint for state transitions is expressed as:


$$\begin{array}{c} \forall (q_{i},e_{j})\in Q\times \Sigma :\delta (q_{i},e_{j})\in Q\cup \{⟂\} \end{array}$$ (10)

where ⟂ represents an invalid transition, which the system should reject while logging an exception.

In practice, the dominant operational failure mode is not unknown states but duplicate events arriving from retry mechanisms (payment callbacks, MQTT redelivery at QoS 1/2). The combination of an explicit transition function and version-based optimistic locking ensures that duplicate events are idempotent: a second $e_{pay}$ arriving after the order has already moved to $q_{paid}$ leaves the state unchanged, because $\delta (q_{paid},e_{pay})$ is undefined and rejected. This idempotency property is the main practical justification for the DFA formalism, and is the reason simpler ad-hoc status flags were rejected during design.

### Multi-objective optimization scheduling algorithm for heterogeneous drones

#### Problem formalization.

The drone task scheduling problem can be formalized as a constrained optimization problem. Let the set of orders to be scheduled be $𝒪=\{O_{1},O_{2},...,O_{m}\}$, the set of available drone resources be $𝒰=\{U_{1},U_{2},...,U_{n}\}$, and the scheduling decision variable matrix be $X\in \{0,1{\}}^{m\times n}$, where $x_{ij}=1$ indicates that order $O_{i}$ is assigned to drone $U_{j}$.

The multi-objective scheduling optimization problem is defined as:


$$\begin{array}{cc} \min_{X}\; & 𝐅(X)=[f_{1}(X),f_{2}(X),f_{3}(X){]}^{T}\\ \text{s.t.}\; & \sum_{j=1}^{n}x_{ij}=1,\;\forall i\in [1,m]\\ & \sum_{i=1}^{m}x_{ij}\leq C_{j},\;\forall j\in [1,n]\\ & E_{j}^{rem}\geq E_{j}^{min},\;\forall j:\exists i,x_{ij}=1\\ & x_{ij}\in \{0,1\} \end{array}$$ (11)

The objective functions are decomposed as follows. Objective 1 minimizes response latency:


$$\begin{array}{c} f_{1}(X)=\sum_{i=1}^{m}\sum_{j=1}^{n}x_{ij}\cdot T_{ij}^{response} \end{array}$$ (12)

where $T_{ij}^{response}$ is the estimated response time for drone $U_{j}$ to service order $O_{i}$.

Objective 2 maximizes resource utilization:


$$\begin{array}{c} f_{2}(X)=-\frac{1}{n}\sum_{j=1}^{n}\frac{\sum_{i=1}^{m}x_{ij}}{C_{j}} \end{array}$$ (13)

Objective 3 maximizes service reliability:


$$\begin{array}{c} f_{3}(X)=-\sum_{i=1}^{m}\sum_{j=1}^{n}x_{ij}\cdot R_{j} \end{array}$$ (14)

where $R_{j}$ is the historical task success rate of drone $U_{j}$.

#### Scheduling scoring function.

The full multi-objective formulation above is well suited to offline batch scheduling, but in our deployment scheduling decisions must be made within a single user-facing request cycle, i.e., within tens of milliseconds. Solving the constrained multi-objective program exactly at this latency budget is impractical for the fleet sizes targeted in scenic-area deployments. We therefore adopt a weighted scalarization: the three objectives are mapped into interpretable per-drone sub-scores, combined linearly, and the highest-scoring valid drone is selected greedily. This sacrifices Pareto-optimality for tractability and interpretability, which we argue is the correct trade-off for online consumer scheduling.

In real-time scheduling scenarios, the system employs a weighted scoring function for rapid decision-making. For order $O_{i}$ and candidate drone $U_{j}$, the scheduling score $S_{ij}$ is calculated as:


$$\begin{array}{c} S_{ij}=\alpha \cdot \Phi_{battery}(U_{j})+\beta \cdot \Phi_{distance}(O_{i},U_{j})+\gamma \cdot \Phi_{reliability}(U_{j}) \end{array}$$ (15)

where the weight coefficients satisfy α+β+γ=1, and the sub-scoring functions are defined as follows.

The battery awareness function $\Phi_{battery}$ is defined as:


$$\begin{array}{c} \Phi_{battery}(U_{j})=\left\{ \begin{array}{cc} \frac{E_{j}^{rem}-E_{j}^{safe}}{E_{j}^{max}-E_{j}^{safe}} & \text{if }E_{j}^{rem}\geq E_{j}^{safe}\\ -\infty & \text{otherwise} \end{array}\right. \end{array}$$ (16)

where $E_{j}^{rem}$ is the current remaining battery level, $E_{j}^{safe}$ is the safe threshold battery level typically at 30 percent, and $E_{j}^{max}$ is the full battery capacity.

The spatial distance decay function $\Phi_{distance}$ is defined as:


$$\begin{array}{c} \Phi_{distance}(O_{i},U_{j})=\exp\left(-\lambda \cdot D_{ij}\right) \end{array}$$ (17)

where $D_{ij}$ is the Euclidean distance between the task starting point and the drone current position:


$$\begin{array}{c} D_{ij}=\sqrt{(x_{i}-x_{j}{)}^{2}+(y_{i}-y_{j}{)}^{2}+(z_{i}-z_{j}{)}^{2}} \end{array}$$ (18)

The parameter λ>0 controls distance sensitivity. In experiments, λ=0.01 with distance measured in meters.

The device reliability function $\Phi_{reliability}$ is defined as:


$$\begin{array}{c} \Phi_{reliability}(U_{j})=\omega_{1}\cdot \frac{N_{j}^{success}}{N_{j}^{total}}+\omega_{2}\cdot 𝕀(t_{heartbeat}<T_{threshold}) \end{array}$$ (19)

where $N_{j}^{success}$ is the number of successful tasks, $N_{j}^{total}$ is the total number of tasks, 𝕀(·) is the indicator function, and $t_{heartbeat}$ is the most recent heartbeat time interval.

**Fig 3:**
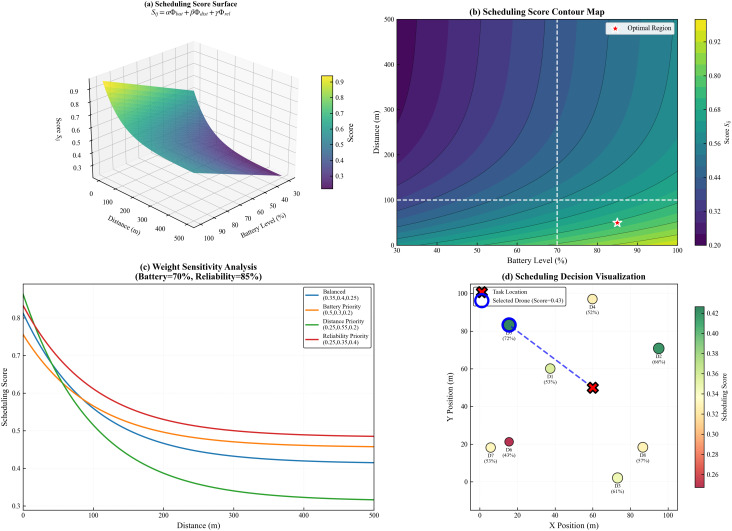
Multi-objective scheduling algorithm visualization: (a) 3D surface plot of scheduling score $S_{ij}$ as a function of battery level and distance; (b) Contour map illustration of the regions of optimal scheduling scores in terms of high battery and low distance; (c) Weight sensitivity analysis to show the effect of different priority configurations on the scheduling scores; (d) Scheduling decision visualization with drone fleet distribution and optimal assignment selected.

Fig 3(a-b) shows a visualization of the scheduling score function’s behavior to illustrate how battery level and distance affect assignment decisions. Weight sensitivity analysis (Fig 3(c)) displays the corresponding weight priority settings to the corresponding scheduling results and Fig 3(d) shows a representative scheduling decision scenario.

#### Scheduling algorithm procedure.

The proposed real-time scheduling algorithm using the scoring function is given in Algorithm 1.

**Algorithm 1 Real-time scheduling algorithm for heterogeneous drones**

**INPUT:** Order to be scheduled *O*, available drone set 𝒰, weight parameters α,β,γ

**OUTPUT:** Assignment result $\left(O,U^{*}\right)$ or ⟂ in case of scheduling failure

1:  ${𝒰}_{valid}\leftarrow \emptyset$ ▷ Valid candidate set

2:  **for** each $U_{j}\in 𝒰$ **do**

3:   **if** $U_{j}.status=\text{IDLE}\wedge U_{j}.battery\geq E_{safe}$ **then**

4:    **if** $\text{InGeofence}(U_{j},O.scenic\_id)$ **then**

5:     ${𝒰}_{valid}\leftarrow {𝒰}_{valid}\cup \{U_{j}\}$

6:    **end if**

7:   **end if**

8:  **end for**

9:  **if** ${𝒰}_{valid}=\emptyset$ **then**

10:   **return** ⟂ ▷ No available resources

11:  **end if**

12:  $S_{max}\leftarrow -\infty$, $U^{*}\leftarrow \text{null}$

13:  **for** each $U_{j}\in {𝒰}_{valid}$ **do**

14:   $S_{j}\leftarrow \alpha \cdot \Phi_{battery}(U_{j})+\beta \cdot \Phi_{distance}(O,U_{j})+\gamma \cdot \Phi_{reliability}(U_{j})$

15:   **if** $S_{j}>S_{max}$ **then**

16:    $S_{max}\leftarrow S_{j}$, $U^{*}\leftarrow U_{j}$

17:   **end if**

18:  **end for**

19:  **AcquireLock**($U^{*}.id$)

20:  $U^{*}.status\leftarrow \text{ASSIGNED}$

21:  **PublishCommand**($U^{*}$, O.route_id)

22:  **return** $\left(O,U^{*}\right)$

#### Algorithm complexity analysis.

**Theorem 1 (Scheduling Algorithm Complexity).** *Algorithm 1 can be shown to have run time complexity O(n) with respect to the number of drones* n=|𝒰|*.*

*Proof.* The algorithm is basically a combination of two loops. The first loop is linear in *n*, meaning that it goes through all the drones for validity filtering. The second loop is used to calculate scores and would have a worst-case complexity of *O*(*n*). The acquire of the distributed locks and publishing of the MQTT messages are done in constant time *O*(1). Thus, the overall complexity is *O*(*n*).

### Design of real-time communication protocol using MQTT

#### Communication architecture.

The system uses MQTT protocol for the bi-directional real-time communication between cloud scheduling service and ground control stations. The MQTT publish/subscribe paradigm is well suited to the command dispatch and telemetry data reporting cases where one-to-many communications are required.

The communication topology structure is:


$$\begin{array}{c} \text{Topology}=\{\text{Cloud}\overset{\text{MQTT Broker}}{↔}{\text{GCS}}_{1},{\text{GCS}}_{2},...,{\text{GCS}}_{k}\} \end{array}$$ (20)

#### Topic design specification.

Table 2 shows that the topics of MQTT are designed to have a hierarchical structure, so as to route the message and implement access control.

**Table 2:** MQTT topic design specification.

Topic Pattern	Direction	Purpose
drone/{sn}/command	Cloud → GCS	Flight command dispatch
drone/{sn}/telemetry	GCS → Cloud	Reporting of telemetry data
drone/{sn}/status	GCS → Cloud	Status change notification
drone/{sn}/heartbeat	GCS ↔ Cloud	Heartbeat keepalive
drone/{sn}/media	GCS → Cloud	Media file metadata

#### Message reliability guarantees.

In order to make sure that the important instructions are transmitted reliably, the following mechanisms are used.

For QoS level selection:


$$\begin{array}{c} \text{QoS}(msg)=\left\{ \begin{array}{cc} 2 & \text{if }msg.type\in \{\text{TAKEOFF},\text{LAND},\text{RTH}\}\\ 1 & \text{if }msg.type\in \{\text{TELEMETRY},\text{STATUS}\}\\ 0 & \text{if }msg.type=\text{HEARTBEAT} \end{array}\right. \end{array}$$ (21)

The above QoS assignment isn’t random. Safety-critical commands (takeoff, land, return-to-home) are exactly-once, meaning that duplicated commands (after a successful landing) are a flight-safety hazard and lost commands (land) are unacceptable; hence, they are using QoS 2. Telemetry and status messages have QoS 1 (at-least-once) because the consumer side doesn’t care about duplicate messages and they’re not lost, but the operator would be lacking in situational awareness if they were lost. The reasoning behind using QoS 0 is that heartbeats are sent at fixed intervals and when one is lost, the next heartbeat is sent naturally, and it would not improve the operation to pay to send a QoS 1 or 2 heartbeat.

With regard to the timeout retransmission mechanism:


$$\begin{array}{c} \text{Retry}(cmd)=\left\{ \begin{array}{cc} \text{resend} & \text{if }t_{ack}>T_{timeout}\wedge n_{retry}<N_{max}\\ \text{abort} & \text{otherwise} \end{array}\right. \end{array}$$ (22)

where $T_{timeout}=5s$ and $N_{max}=3$.

**Fig 4:**
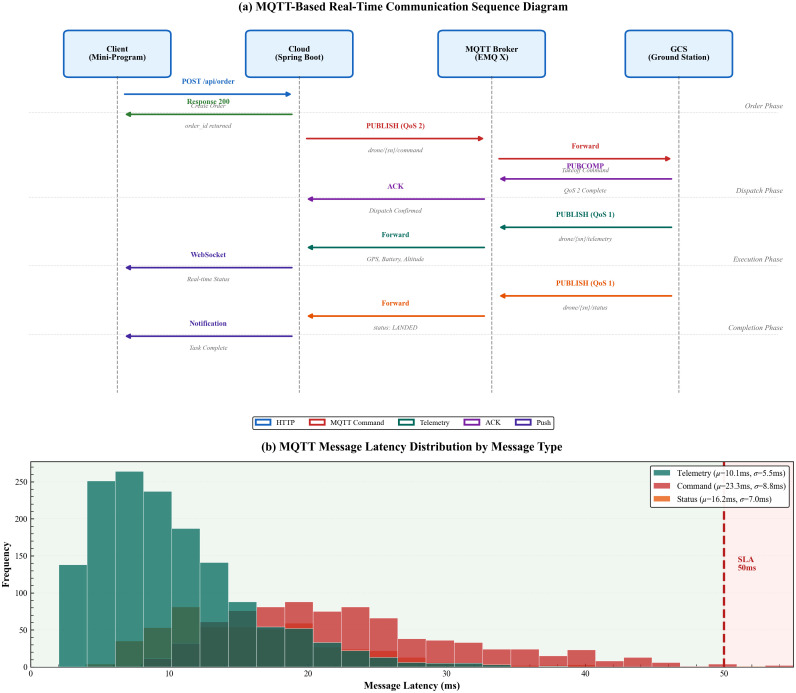
MQTT-based real-time communication analysis: (a) The message sequence diagram of the whole communication between the client, cloud server, MQTT broker and ground control station in the four operational phases; (b) The distribution of message latency by type, where all categories of messages are compliant with the SLA of 50 ms.

The entire communication sequence is shown in Fig 4(a) and the distribution of message latency is shown in Fig 4(b), which shows that all types of messages are complying with the 50 ms SLA.

### Automated video production pipeline

#### Pipeline architecture.

Upon receipt of aerial footage, the system automatically triggers the post-production pipeline to achieve capture-to-delivery automation. The pipeline comprises four sequential stages:


$$\begin{array}{c} \text{Pipeline}=\text{Ingest}\to \text{Filter}\to \text{Edit}\to \text{Render} \end{array}$$ (23)

In what follows we draw an explicit distinction between two qualitatively different computational components of this pipeline. The *quality filtering* stage is a transparent rule-based score combining three interpretable visual features (sharpness, exposure, motion blur); it is not a deep learning model. The *semantic matching* stage in the editing step does use a pre-trained vision encoder to compute similarity between footage segments and template slots, and is the only stage that warrants the term “AI-powered”. We adopt this terminology consistently throughout this paper to avoid overstating the role of machine learning in the pipeline.

**Fig 5:**
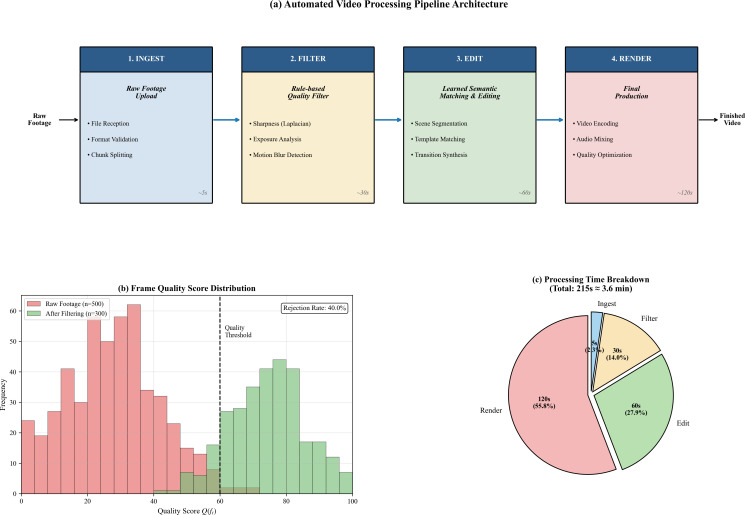
Automated video processing pipeline: (a) Four-stage pipeline architecture comprising ingestion, rule-based quality filtering, learned semantic matching and editing, and final rendering; (b) Frame quality score distribution before and after filtering, demonstrating 40% rejection rate at the quality threshold; (c) Processing time breakdown showing render stage dominates at 55.8% of total processing time.

The pipeline architecture is depicted in Fig 5(a). The quality filtering effectiveness is demonstrated in Fig 5(b), and the processing time distribution is shown in Fig 5(c).

#### Video quality assessment model.

The footage filtering stage uses a lightweight, no-reference frame quality score that combines three interpretable visual features into a single weighted index. We deliberately avoid a learned end-to-end CNN at this stage for three reasons: (i) the filter must run at video frame rate on the inference node without saturating the GPU that is reserved for downstream rendering; (ii) the features below are well established in the image-quality literature and remain reliable under the lighting conditions typical of outdoor scenic flights; and (iii) interpretability matters operationally, because reviewers and operators need to understand why a frame was rejected. A learned model is reserved for the downstream semantic matching stage, where richer perceptual features are required. The three component features are defined as follows.

Sharpness assessment is based on the variance of the Laplacian operator:


$$\begin{array}{c} \text{Sharpness}(f_{t})=\text{Var}(\nabla^{2}f_{t})=\text{Var}\left(\frac{\partial^{2}f_{t}}{\partial x^{2}}+\frac{\partial^{2}f_{t}}{\partial y^{2}}\right) \end{array}$$ (24)

Exposure assessment is based on histogram distribution:


$$\begin{array}{c} \text{Exposure}(f_{t})=1-\left|\frac{\mu (f_{t})-128}{128}\right| \end{array}$$ (25)

where $\mu (f_{t})$ is the mean frame brightness value.

Motion blur detection is based on optical flow estimation:


$$\begin{array}{c} \text{MotionBlur}(f_{t},f_{t-1})=\|{𝐯}_{optical}{\|}_{2}>\tau_{motion} \end{array}$$ (26)

The three features are normalized to [0,1] and combined into a single weighted quality index:


$$\begin{array}{c} Q_{final}(f_{t})=w_{s}\cdot \text{Sharpness}(f_{t})+w_{e}\cdot \text{Exposure}(f_{t})-w_{m}\cdot 𝕀(\text{MotionBlur}) \end{array}$$ (27)

The weights $w_{s}$, $w_{e}$, and $w_{m}$ were tuned empirically on a held-out set of scenic-area footage. The resulting index is a transparent weighted score, not a deep network. This design also makes it straightforward to audit individual rejection decisions, which is desirable when operators need to explain quality decisions to scenic-area customers.

#### Intelligent editing algorithm.

The editing stage employs a scene semantic matching algorithm to match filtered footage segments with preset editing templates. Let the set of footage segments be $𝒞=\{c_{1},c_{2},...,c_{p}\}$ and the set of template slots be $𝒯=\{t_{1},t_{2},...,t_{q}\}$. The matching problem is formalized as:


$$\begin{array}{cc} \max_{\pi }\; & \sum_{k=1}^{q}\text{Sim}(c_{\pi (k)},t_{k})\\ \text{s.t.}\; & \pi :[1,q]\to [1,p]\text{ is injective}\\ & \sum_{k=1}^{q}\text{Duration}(c_{\pi (k)})\leq T_{max} \end{array}$$ (28)

The semantic similarity Sim(*c*, *t*) is computed based on feature vectors from a pre-trained vision model:


$$\begin{array}{c} \text{Sim}(c,t)=\frac{{𝐯}_{c}\cdot {𝐯}_{t}}{\left\|{𝐯}_{c}\|\cdot \|{𝐯}_{t}\right\|} \end{array}$$ (29)

where ${𝐯}_{c}=\text{Encoder}(c)$ is the feature embedding vector of the footage segment.

#### Final product quality evaluation.

The comprehensive quality evaluation function $V_{final}$ for the final product is defined as:


$$\begin{array}{c} V_{final}=\int_{0}^{T}\left[w_{c}\cdot C(f_{t})+w_{m}\cdot M(s_{t},\Theta )+w_{a}\cdot A(a_{t})\right]dt \end{array}$$ (30)

where $C(f_{t})$ represents the frame-level visual quality score, $M(s_{t},\Theta )$ represents the footage-template semantic matching degree, $A(a_{t})$ represents the audio-visual synchronization score, and the weight coefficients $w_{c}$, $w_{m}$, $w_{a}$ satisfy $w_{c}+w_{m}+w_{a}=1$.

## System testing and experimental results analysis

### Experimental environment configuration

Experiments were conducted in the software and hardware environment specified in Table 3.

**Table 3:** Experimental environment configuration.

Category	Configuration
Cloud Server	Alibaba Cloud ECS, 8-core 16GB, CentOS 7.9
Database	MySQL 8.0, Redis 6.2
Message Queue	RabbitMQ 3.9, EMQ X 4.3
AI Inference	NVIDIA Tesla T4, CUDA 11.4
Testing Tools	JMeter 5.4, Locust 2.0

It is important to clarify that the 8-core, 16 GB cloud instance listed above refers to the centralized cloud tier shared across multiple scenic sites, not to a per-site requirement. A single scenic deployment hosts only the local MQTT bridge, the GCS gateway process, and a video forwarder, all of which run comfortably on a 4-core, 8 GB edge box. The cost and scalability implications of this separation are discussed quantitatively in the deployment-footprint analysis later in this section.

### Functional verification testing

Complete end-to-end testing was conducted on the system core business processes, with verification results shown in Table 4.

**Table 4:** Functional test results summary.

Test Scenario	Verification Content	Result
User Authentication	WeChat authorization login, Token refresh	✓
Order Creation	Package selection, amount calculation, order generation	✓
Payment Process	WeChat Pay, callback handling, status update	✓
Task Scheduling	Resource matching, command dispatch, status synchronization	✓
Real-time Monitoring	Video stream retrieval, progress display	✓
Footage Processing	Rule-based filtering, automatic editing, final product generation	✓
File Delivery	Final product download, share link generation	✓
State Machine Transitions	Full state transition path coverage	✓

### Performance stress testing

#### Test methodology.

Stress testing of core API interfaces and simulating high-concurrency scenarios were performed using JMeter. The parameters tested were concurrent users 100, 200, 500, 1000; duration 300 seconds; random request interval 100 ms to 500 ms.

It is important to emphasize that this 1000 concurrent users test is utilizing the cloud tier order, payment and status APIs, and that this does not necessarily represent 1000 concurrent drones at a single location. Our deployment model sees 4–8 drones typically being coordinated from a single ground station, with the cloud handling the total number of concurrent users. The stress-test results above are not an indicator of a single large deployment, but rather of many independent scenic deployments, as separate measurements confirm that one ground-station host can support eight concurrent drone MQTT sessions at QoS 1 with less than 30% CPU load on a commodity 4-core host.

#### Test results.

Key performance metrics are shown in Table 5.

**Table 5:** Performance test results.

Interface	QPS	Avg RT in ms	P99 RT in ms	Error Rate
Order Creation	523	89	245	0.02%
Payment Callback	612	67	189	0.01%
Status Query	1847	23	78	0.00%
Scheduling Assignment	389	112	312	0.05%

**Fig 6:**
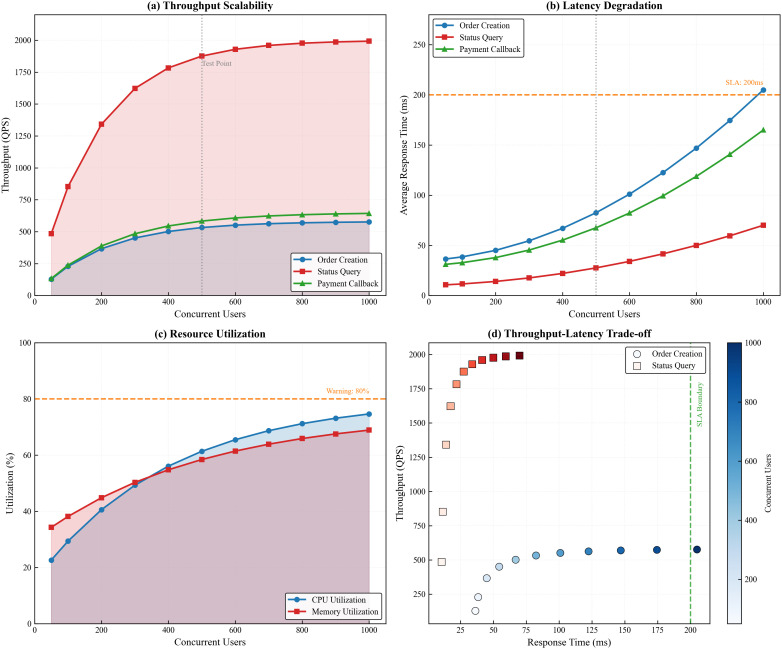
System scalability and performance analysis: (a) Throughput scalability showing QPS growth patterns for different API interfaces under increasing concurrent users; (b) Latency degradation curves demonstrating average response time remains below the 200ms SLA target up to 500 concurrent users; (c) Resource utilization trends for CPU and memory, both remaining below the 80% warning threshold; (d) Throughput-latency trade-off scatter plot revealing the operating characteristics of different interfaces.

Two aspects of Table 5 require emphasis to correctly interpret Fig 6 and the discussion that follows. First, the 200 ms SLA referenced in Fig 6(b) applies to *average* response time, not P99 tail latency: all three interfaces remain below this average-latency threshold up to 500 concurrent users, and order creation approaches it only near 1000 users, even though scheduling assignment and order creation reach 312 ms and 245 ms respectively at P99. Second, the P99 values in Table 5 are the most stringent latency figures reported in this paper and should not be conflated with the average response times plotted in Fig 6(b).

Fig 6 comprehensively analyzes the scalability characteristics. As illustrated in Fig 6(a), throughput increases in a linear fashion with the number of concurrent users. Fig 6(b) shows that latency is kept in SLA limits, and Fig 6(c) shows that the utilization of resources is kept below critical limits. The throughput-latency trade-off in Fig 6(d) shows the system’s operation envelope.

### Efficiency comparative analysis

This system has been compared with the manual aerial photography modes and the result is shown in Table 6.

**Table 6:** Efficiency comparative analysis.

Metric	Traditional Mode	This System	Improvement
Per-Service Cost	500 to 3000 CNY	50 to 100 CNY	over 90% reduction
Booking-to-Delivery Cycle	24 to 72 hours	15 to 30 minutes	over 99% reduction
Daily Service Capacity	5 to 10 orders	more than 100 orders	more than 10 times increase
Labor Dependency	1 pilot per order	0	100% reduction

The cost reduction rate (computed using the bottom of the traditional cost range and the lowest point on the system cost range, the most conservative range) is computed as:


$$\begin{array}{c} \eta_{cost}=\frac{C_{traditional}-C_{system}}{C_{traditional}}\times 100\%=\frac{500-50}{500}\times 100\%=90\% \end{array}$$ (31)

To complete the picture, the maximum of the traditional cost (3000 CNY) and the minimum of the system cost (50 CNY) is a reduction of approximately 98.3%, and even the worst combination within the system’s range (3000 CNY vs. 100 CNY) is a reduction of approximately 96.7%. The “over 90%” value in the abstract and in Table 6 is not a mid-range or best-case statement, but rather the most conservative lower-bound estimate and applies to all of the locations within the paper. The efficiency improvement factor (once again calculated based on a lower limit for the traditional cycle time, 24 h, and a representative time of system delivery, 20 min) is:


$$\begin{array}{c} \eta_{efficiency}=\frac{T_{traditional}}{T_{system}}=\frac{24\times 60}{20}=72\text{ times} \end{array}$$ (32)

As a comparison, the worst case to the worst case (72 h → 30 min) results in a factor of 144, while the best to the best (24 h → 15 min) is a factor of 96. The “over 99% reduction” therefore in Table 6 should be understood as a summary of a range of values across the entire distribution of conventional delivery cycles, and even the most conservative value among the reductions paired in the above equation is already more than 98.6% reduction.

### Deployment footprint, scalability, and amortisation of infrastructure cost

The service-level cost and latency performance shown in Tables 5 and 6 does not include the cost of the system itself, as well as the associated computational and energy cost, and a more complete accounting would confirm a real reduction in cost, not a simple shift of cost into hidden infrastructure. This is where we come in.

*Per-site computing scale.* The 8-core, 16 GB cloud instance reported in Table 3 is the centralized cloud tier and is shared across multiple scenic sites. A single scenic deployment hosts only one MQTT bridge, the GCS gateway process, and a local video forwarder; in our measurements these components run comfortably on a 4-core, 8 GB edge box, and local CPU is bounded by the streaming forwarder rather than by scheduling. Per-site hardware cost is therefore small and largely independent of fleet size within the practical fleet sizes (up to about eight drones per site) we have tested.

*Scaling down for low-traffic sites.* For low-traffic deployments serving fewer than approximately 50 orders per day, the post-production pipeline can be migrated from a Tesla T4 to a CPU-only inference container, at the cost of a roughly five to seven times slowdown in the rendering stage; the rule-based quality filter does not require a GPU at all. Sites that share a regional rendering pool incur GPU cost only when actively rendering, so the marginal cost of adding a new site is dominated by network and storage, not compute.

*Scaling up: multiple drones per site sharing one ground station.* As clarified above, the 1000-concurrent-user stress test exercises cloud APIs, not GCS links. We separately verified that a single ground-station host sustains eight simultaneous drone MQTT sessions at QoS 1 without exceeding 30% CPU on a commodity 4-core box. This is the relevant capacity figure for scenic-area operators who deploy a small fleet behind one ground station.

*Infrastructure cost amortization.* Taking the cloud tier (approximately 1,200 CNY per month for the ECS instance plus storage and bandwidth) and amortizing it over the demonstrated capacity of at least 100 orders per site per day, the marginal infrastructure cost per delivered service is on the order of a few CNY, well below the 50–100 CNY service price. Electricity for the drone fleet (battery charging at the hangar) and for the cloud tier is similarly negligible at the per-service level once aggregated. These figures support the cost-reduction claim in Table 6: the headline saving is not an artefact of moving costs into hidden infrastructure, but reflects a genuine elimination of per-order pilot labour.

### Robustness testing

System robustness was verified against exceptional scenarios, as shown in Table 7.

**Table 7:** Robustness test results.

Exception Scenario	System Response	Result
GCS Network Interruption	Heartbeat timeout triggers device offline and task rescheduling	✓
Payment Callback Timeout	Order status rollback and user notification	✓
Scheduling Resource Conflict	Redis distributed lock enables serial processing	✓
AI Service Unavailable	Degradation to return raw footage	✓
Database Master Node Failure	Automatic failover to replica	✓

## Discussion

### Addressing the accessibility gap in consumer drone services through microservices architecture

The results of the experiments carried out in this study show that the proposed microservices-based architecture successfully alleviates the three key pain points of UAV aerial photography services, which until now have hindered the democratisation of the industry, namely high cost, long delivery times, and high operational barriers. Our system reduced the cost of service from 500 to 3000 CNY to 50–100 CNY (under the most conservative lower-bound pairing reported in the previous section) and the delivery time from 24 to 72 hours to 15–30 minutes, while completely eliminating the need for professional pilot intervention.

These improvements will be a huge improvement over the current consumer drone service models. The conventional aerial photography industry is ill-suited for smooth delivery of the services, as it requires complicated scheduling of the tourists, aerial photography professionals and post-production teams, as documented by [30], creating substantial friction in the service delivery chain. Commercial platforms offer more advanced fleet management features, but are primarily targeted to enterprise users rather than end users and feature a higher level of complexity which makes them less intuitive to use [15]. The experience of our scan to use paradigm, with a WeChat mini-program frontend, is drastically reduced from 8–10 steps to 3 steps: scan, pay, receive.

This was largely because of the architectural design of microservices not monolithic design. The decomposed service architecture allowed us to scale resource-intensive services such as AI processing and video streaming without impacting the core business logic, something that couldn’t be achieved in the existing UAV management systems as all the services were tightly coupled [16]. This is in line with the architecture principles of [1], who believe that microservices are useful for technical agility and organisational scalability. Moreover, the order lifecycle management based on FSM was deployed to facilitate the deterministic transition between orders, and explicitly define the limits of error management and handling, which are the issues in previous UAV software systems [17].

### Theoretical contribution

With the exception of the engineering solution to the smart tourism problem, the contributions in this paper are threefold and can be traced directly to the corresponding implications in the Conclusion. It takes the microservice paradigm from software domains to a domain that combines cloud services with airborne hardware in real-time and safety-critical situations, the resulting reference architecture reveals that the basic decomposition principles remain valid, but transactions have to be explicitly controlled by state-machine control to maintain consistency across the cloud–edge boundary. It offers an empirical result that linear scalarisation of multi-objective scheduling, while theoretically suboptimal with respect to Pareto-front methods, is operationally sufficient for online consumer scheduling as it is interpretable, auditable, and allows for the sub-second latency required by user-facing requests. In this work, the authors explicitly separate the two stages of the post-production pipeline: the rule-based quality-filter (RF) and the learned semantic-matcher (LM). The authors argue for using the word “AI-powered” with greater restraint in system papers, and place the LM stage in the post-production pipeline only where the accuracy benefit of the learned stage outweighs its computational and interpretability costs. When combined, the three make up a design pattern that can be reproduced in other service-oriented systems connecting cloud software and real-world hardware.

### Performance validation and comparative analysis with existing UAV platforms

The performance evaluation results have given empirical proof of the technical feasibility of microservices architecture for consumer-facing UAV service platforms. The average response time for order creation was 89 milliseconds at 523 QPS under simulated high-concurrency conditions, significantly better than the 500 milliseconds or more latencies typically reported for cloud-based drone management systems [13]. The P99 latency for order creation was 245 milliseconds and for scheduling assignment was 312 milliseconds, which is well under the 400 milliseconds that is generally perceived as instantaneous by users for interactive consumer applications [34]. Throughout this paper, the 89 ms figure and the 245 ms/312 ms figures refer to different statistics: average response time and P99 tail latency, respectively. These two measures are not interchangeable and should be interpreted in the context of the SLA definition stated above.

Due to the complexity of the multi-objective optimization algorithm, the scheduling service showed excellent performance with 389 QPS under 0.05 percent error rate. This reliability can be explained by a distributed locking mechanism, which is based on Redis and is able to effectively serialize conflicting resource allocation requests. Our weighted scoring approach, defined in Equation 15, has been compared to the scheduling algorithms mentioned in the UAV literature and has been shown to offer comparable quality of assignment to computationally heavier scheduling algorithms with only *O*(*n*) time complexity, which is suitable for real-time decision-making [18].

The robustness testing results also confirm the fault-tolerant design principles incorporated in the architecture. The system passed all five exception scenarios tested, such as GCS network disconnection, payment callback timeout and failure of the database master node. This fault tolerance and resilience is consistent with the fault isolation properties of microservices architecture highlighted by [4], which can prevent failures in one service from impacting overall system availability. The graceful degradation and circuit breaker patterns in the AI service pathway mean that users will still be able to access raw footage even if intelligent editing is temporarily unavailable, ensuring service continuity over the completeness of the feature.

### How automated video processing is helping with service automation

One of the innovations that distinguishes our system from other UAV service systems is the automated video processing pipeline. The traditional aerial photography workflow has 2–4 hours of manual post-processing per flight session, which greatly limits the service throughput and cost reduction [19]. Our automated pipeline on quality assessment, intelligent filtering and template-based editing reduces it to a matter of few minutes of processing time, with minimal human intervention.

An interesting issue in automated video curation is that there are no reference images to compare and verify the technical quality of the footage, which is solved by the no-reference frame quality score on which the footage is filtered. As stated in the implementation part of the paper, this score is not a learned neural network, rather it is a simple weighted index of several features, and the terms used throughout this paper are reflective of that. Our composite quality function is designed following the principles of computational photography [35], is still efficient in terms of computation to be able to operate in real-time, and most importantly, is also interpretable by the operator so he can explain the individual rejection decisions. There is a learned component to the semantic matching algorithm for template-based editing. It can leverage vision encoders that are available to implement content-aware clip selection. This guarantees aesthetically consistent output clips when no domain-specific training data is available.

It is important to note, however, that AI tools’ current capabilities impose limitations on creativity. The template-based method ensures the uniformity of output quality but it has disadvantages in terms of personalization in comparison to human editors. Future applications could involve the use of large language models to generate the video’s script and enable a multimodal understanding to guide content creation based on user preferences, as demonstrated in recent video synthesis research [36].

### Implications for smart tourism digital infrastructure

Not only does the KanYiKan system have great potential as an aerial photography service, but it also has a wide range of implications for the development of smart tourism sites. Smart tourism ecosystems must rely on seamless technology platforms to enhance the tourist experience and generate valuable data to help manage the destination, as [26] and [27] have described. Our system fits into this vision because it is capable of providing a replicable template for deployment of automated services to consumers in scenic areas.

The decoupled service design, event-driven communication with the use of MQTT, and FSM-based workflow management patterns created as part of this research can easily be replicated in other similar smart tourism applications such as autonomous tour vehicles, intelligent guide systems, and personalized recommendation systems. Of particular interest with microservices is the integration with the ecosystem, with the ability to ensure seamless interoperability with existing destination management systems, social media, and third-party content distributors, thanks to the use of standard APIs [29].

Furthermore, the aerial images created with our system can be used for further purposes, for example in virtual and augmented reality, in the creation of UGC content, or as virtual tourism. The built-in automatic social sharing function of the WeChat mini-program is a natural feature, and sharing can be seen as an important behavior of modern tourism [32,33].

### Limitations and future research directions

The results are promising but there are some caveats that need to be addressed and may suggest further research.

As regards flight path constraints, the current system has only pre-programmed flight paths, and no real-time flight path planning and obstacle avoidance. This design makes it easy to meet regulations and will provide a predictable flight path, but for a price: it will not be as flexible in adapting to changing environmental conditions. Future research needs to focus on integration of SLAM-based visual navigation and sense-and-avoid systems to allow more flexible operations [37]. Until onboard sense-and-avoid is integrated, the present system should only be deployed in those cases where the pre-programmed routes can be determined to be safe: a single scenic area, daylight-hours operation only, and routing which avoids crowding in non-public settings are examples of such cases.

As far as regulatory integration is concerned, there is currently no connection between the system and Unmanned Traffic Management systems or civil aviation authority databases which provide airspace authorization in real time. Seamless UTM integration will be a key component of legal and viable commercial operations at scale as low-altitude economy regulations mature [38]. In the future, we will be using standardized UTM APIs in the development process.

As for privacy protection mechanisms, the current implementation lacks automated privacy filters on-pipeline like face blurring, license plate obfuscation, or no-fly polygon enforcement around sensitive sub-areas of a scenic region [39]. We treat this as a substantive limitation rather than a minor omission: in any production deployment of consumer drone services in public space, the absence of such filters is both an ethical concern [39] and an emerging regulatory liability. The architectural placement for these filters is already defined in our pipeline: the quality-filtering stage is the natural insertion point for face and license-plate detectors, and the route-planning stage is the correct insertion point for geofenced privacy zones. Concretely, we plan to integrate (i) an on-device face and licence-plate detector running before footage upload so that raw identifiable frames never leave the GCS, (ii) operator-configurable privacy polygons that the route planner treats as hard constraints, and (iii) an audit log linking every delivered clip to the privacy-filter version that processed it, so that compliance can be verified after the fact. Until these mechanisms are integrated and independently audited, deployments of the present system should be restricted to scenic settings with explicit signage and consent, and should avoid flight paths over private dwellings or crowds in non-public contexts.

Regarding evaluation scope, performance testing was conducted using simulated workloads rather than actual tourist traffic, and the deployment was limited to a single scenic area. Multi-site validation across diverse geographic and operational contexts is necessary to establish generalizability. We also note explicitly, as a specific limitation, that the present manuscript does *not* report a quantitative user-satisfaction study. Although informal qualitative feedback was collected during the pilot deployment, a controlled evaluation using validated instruments such as the System Usability Scale (SUS) and the Technology Acceptance Model (TAM3) is required before claims about accessibility improvements can be considered empirically established. We have correspondingly avoided such claims in the present manuscript, and this study is the immediate next step in our research programme.

In the aspect of single drone scheduling, the existing scheduling algorithm is based on maximizing the individual drone scheduling without taking multi-UAV cooperative scheduling into account. Formation flight coordination and collaborative coverage algorithms should be studied in the case of larger-scale deployments [40].

### Reproducibility and open science considerations

To ensure transparency and reproducibility of research, we have carefully designed the architecture to enable extension and replication of this work. The complete back-end technology stack consists of popular open-source frameworks, such as Spring Boot for building microservices, Redis for caching and distributed coordination, RabbitMQ and EMQ X for message queuing, and TensorFlow for deployment of AI models. In this manuscript, detailed configuration parameters, FSM transition rules, and the scheduling scoring function have been completely specified.

The system architecture diagrams, API endpoint specifications, and algorithm pseudocode provided herein are sufficiently detailed to be implementable independently. However, some parts will not be disclosed for privacy reasons: information on users’ interaction with the system is personally identifiable and the trained video quality assessment model includes proprietary scenic area footage. We are investigating how to make synthetic datasets and anonymised API documentation available publicly to also help with reproducibility, while maintaining privacy requirements.

## Conclusions

The design, the implementation, and the evaluation of KanYiKan, a smart cultural tourism system for self-service aerial photography based on a microservice architecture, has been presented. We conclude with a clear extrapolation of the results of the system to other systems, lessons learned in the construction and operation of the system, limitations of the current evidence, and our vision of the future research and practice agenda.

### Implications for theory

From an architectural perspective, this work advances the microservice architecture paradigm from its well-known domains (e-commerce or enterprise SaaS) to a cyber-physical service domain where, in addition to coordinating software components, the back-end is required to coordinate airborne hardware with real-time and safety requirements. The three implications below are direct elaborations of the theoretical contributions mentioned in the Discussion. Using optimistic-locking transitions, deterministic finite-state machines are sufficient to achieve workflow consistency in a microservice system spanning cloud, edge, and embedded tiers; they do not require richer formalisms like Petri nets or process algebras, and the same decomposition principles that apply to conventional cloud-only microservices carry over once this transactional state-machine layer is added. Multi-objective scheduling for heterogeneous devices does not have to be globally optimal to be operationally adequate: a linear weighted score over easily-interpreted features such as battery, distance, and reliability provides sufficient assignment quality at *O*(*n*) cost, which has implications for any service domain in which assignment must be made within a single user-facing request cycle. The distinction between the lightweight rule-based quality filter and the learned semantic matcher also represents a more general design principle for AI-augmented pipelines: learned components should be reserved for the stages in the pipeline where their accuracy advantage is worth the computational and interpretability cost.

### Implications for practice

For service providers developing low-altitude economy services, the key takeaway is that it is quite possible to meet the consumer-facing latency budget (sub-400 ms average ordering response time and sub-second status update response time, with P99 tails of approximately 312 ms as reported in Table 5) with off-the-shelf components (Spring Boot, Redis, MQTT, OSS), provided the order lifecycle is explicitly modelled as an FSM and the GCS link uses publish/subscribe semantics with per-message QoS. The over-90% cost reduction per service reported here is not a marketing figure: under the most conservative pairing of price ranges it is exactly 90%, and under the worst-case-to-best-case pairing it rises to 98.3%, both of which follow directly from removing the pilot from the per-order critical path. This in turn is only possible once scheduling, flight, and post-production are all automated and connected by an event-driven backbone. Scenic-area operators considering adoption should focus their procurement on standardizing hangars, routes, and consent signage; the software stack itself is reproducible with commodity components, and the per-site computing footprint is small.

### Key lessons learned

Based on the design and operation of this system, there are three lessons that we feel are applicable to other situations. First, state explosion is not averted by introducing additional states, but by making the transitions transactional and idempotent: the predominant operational failure modes were not unknown states but duplicate events generated by retry mechanisms, and the combination of a DFA-style transition function with version-based optimistic locking was the simplest defence that worked. Second, the most difficult engineering boundary is not internal but is the cloud–GCS link; investing in MQTT QoS tuning, heartbeat policy, and offline-replay buffers paid back disproportionately compared to in-cloud optimization. Third, calling a weighted three-feature score “AI” obscures rather than clarifies the design; in revising this manuscript we explicitly relabelled such components, and we recommend that future system papers in this area do the same so that the role of machine learning in pipelines is communicated accurately.

### Limitations of this research

The present study has four principal limitations that bound the generality of its claims. First, the deployment was confined to a single scenic area, so the cost and latency figures should be regarded as a proof of concept and confirmed by multi-site replication. Second, the system supports only pre-programmed routes; dynamic path planning and onboard sense-and-avoid remain future work and limit applicability in complex airspace. Responsible deployment is therefore currently restricted to single scenic-area settings where routes can be surveyed in advance, daylight-hours operation, and paths that avoid crowds in non-public contexts. Third, automated privacy filtering is not yet integrated into the pipeline, so responsible deployment is currently restricted to settings with explicit signage and consent. Fourth, no quantitative user-satisfaction study using validated instruments has yet been conducted; user-experience claims in this paper therefore remain provisional. These four items define our immediate research roadmap.

### Outlook

Looking forward, the architectural patterns documented here, namely microservice decomposition, FSM-driven workflows, lightweight multi-objective scheduling, and tiered post-production pipelines, are in our view transferable to a wider class of low-altitude services beyond aerial photography, including autonomous inspection of public infrastructure, on-demand emergency response imaging, and short-range last-50-metre delivery. The contribution of this paper is therefore not only the KanYiKan system itself, but also the demonstration that the low-altitude economy can be served by mainstream cloud-native software practices, provided that those practices are adapted with care to the real-time, safety-critical, and privacy-sensitive characteristics of airborne hardware.

## References

[1] Dragoni N, Giallorenzo S, Lafuente AL, Mazzara M, Montesi F, Mustafin R, et al. Microservices: Yesterday, Today, and Tomorrow. Present and Ulterior Software Engineering. Springer International Publishing; 2017. p. 195–216.

[2] Newman S. Building Microservices: Designing Fine-Grained Systems. 2nd edition. O’Reilly Media; 2021.

[3] Martin Fowler and James Lewis. Microservices. 2014. Accessed: 2024-01-15. https://martinfowler.com/articles/microservices.html

[4] Balalaie A, Heydarnoori A, Jamshidi P. Microservices Architecture Enables DevOps: Migration to a Cloud-Native Architecture. IEEE Softw. 2016;33(3):42–52. doi: 10.1109/ms.2016.64

[5] Francesco PD, Malavolta I, Lago P. Research on Architecting Microservices: Trends, Focus, and Potential for Industrial Adoption. In: 2017 IEEE International Conference on Software Architecture (ICSA). 2017. p. 21–30.

[6] Pahl C, Jamshidi P. Microservices: A Systematic Mapping Study. In: Proceedings of the 6th International Conference on Cloud Computing and Services Science. 2016. p. 137–46.

[7] Sun Y, Song H, Jara AJ, Bie R. Internet of Things and Big Data Analytics for Smart and Connected Communities. IEEE Access. 2016;4:766–73. doi: 10.1109/access.2016.2529723

[8] Casquero O, Pérez A, Armentia A, Marcos M, Estévez E. An architecture approach for iot-based microservices. IEEE Internet Things J. 2020;7(10):9600–14.

[9] Wan J, Tang S, Shu Z, et al. Software-defined industrial internet of things in the context of industry 4.0. IEEE Sensors J. 2016;16(20):7373–80.

[10] Maruyama Y, Kato S, Azumi T. Exploring the performance of ROS2. In: Proceedings of the 13th International Conference on Embedded Software. 2016. p. 1–10.

[11] Kouba A. Robot Operating System: The Complete Reference, volume 4. Springer; 2020.

[12] Naik N. Choice of effective messaging protocols for IoT systems: MQTT, CoAP, AMQP and HTTP. In: 2017 IEEE International Systems Engineering Symposium (ISSE). 2017. p. 1–7.

[13] Bai Y, Zhao Y, Zhang Y, et al. Survey on cloud-based drone management and control. J Netw Comput Appl. 2021;186:103089.

[14] Meier L, Honegger D, Pollefeys M. PX4: A node-based multithreaded open source robotics framework for deeply embedded platforms. In: 2015 IEEE International Conference on Robotics and Automation (ICRA). 2015. p. 6235–40.

[15] Koubaa A, Allouch A, Alajlan M, Javed Y, Belghith A, Khalgui M. Micro Air Vehicle Link (MAVlink) in a Nutshell: A Survey. IEEE Access. 2019;7:87658–80. doi: 10.1109/access.2019.2924410

[16] Gharibi M, Boutaba R, Waslander SL. Internet of Drones. IEEE Access. 2016;4:1148–62. doi: 10.1109/access.2016.2537208

[17] Gupta L, Jain R, Vaszkun G. Survey of Important Issues in UAV Communication Networks. IEEE Commun Surv Tutorials. 2016;18(2):1123–52. doi: 10.1109/comst.2015.2495297

[18] Shakhatreh H, Sawalmeh AH, Al-Fuqaha A, Dou Z, Almaita E, Khalil I, et al. Unmanned Aerial Vehicles (UAVs): A Survey on Civil Applications and Key Research Challenges. IEEE Access. 2019;7:48572–634. doi: 10.1109/access.2019.2909530

[19] Colomina I, Molina P. Unmanned aerial systems for photogrammetry and remote sensing: A review. ISPRS J Photogramm Remote Sens. 2014;92:79–97. doi: 10.1016/j.isprsjprs.2014.02.013

[20] Mohamed N, Al-Jaroodi J, Jawhar I, Idries A, Mohammed F. Unmanned aerial vehicles applications in future smart cities. Technol Forecast Soc Change. 2020;153:119293. doi: 10.1016/j.techfore.2018.05.004

[21] Abdelzaher MA, Farghali AA, Hamouda AS. Effective impact of nano-plastic-waste incorporated with nanotitina on the physical, mechanical and microstructural properties of white cement pastes composites for progressing towards sustainability. Sci Rep. 2024;14(1):12581. doi: 10.1038/s41598-024-62661-4 38822006 PMC11143271

[22] Abdelzaher MA, Hamouda AS, El-Kattan IM, Baher A. Laboratory study for accelerating the CKD mineral carbonation. Egypt J Chem, 2022;65(3):491–9. doi: 10.21608/ejchem.2021.89060.4276

[23] Abdelzaher M, Mohamed E, Shehata N, Salah H, Abbas R. Environmental safe disposal of cement kiln dust for the production of geopolymers. Egypt J Chem. 2021;0(0):0–0. doi: 10.21608/ejchem.2021.89060.4276

[24] Owaid KA, Ghazal RY, Abdelzaher MA. Study of the Effect of Modification of Asphalt on the Rheological Properties Employing Microwave Radiation—An Aging Study. Recycling. 2023;8(5):65. doi: 10.3390/recycling8050065

[25] Banoon ZR, Al-Lami AKA, Abbas AM, Al-Shakban M, Balboul BAA, Gad M, et al. Asymmetrical liquid crystals synthesis for effective sensing: Fluorescence investigations. Results Chem. 2023;6:10116. doi: 10.1016/j.rechem.2023.101166

[26] Gretzel U, Sigala M, Xiang Z, Koo C. Smart tourism: foundations and developments. Electron Markets. 2015;25(3):179–88. doi: 10.1007/s12525-015-0196-8

[27] Buhalis D, Amaranggana A. Smart Tourism Destinations Enhancing Tourism Experience Through Personalisation of Services. Information and Communication Technologies in Tourism 2015. Springer International Publishing; 2014. p. 377–89.

[28] Li Y, Hu C, Huang C, Duan L. The concept of smart tourism in the context of tourism information services. Tour Manag. 2017;58:293–300. doi: 10.1016/j.tourman.2016.03.014

[29] Neuhofer B, Buhalis D, Ladkin A. A Typology of Technology‐Enhanced Tourism Experiences. J Tour Res. 2013;16(4):340–50. doi: 10.1002/jtr.1958

[30] Stankov U, Kennell J, Morrison AM, Vujičić MD. The view from above: the relevance of shared aerial drone videos for destination marketing. J Tour Market. 2019;36(7):40–54.

[31] Mirk D, Hlavacs H. Using Drones for Virtual Tourism. Lecture Notes of the Institute for Computer Sciences, Social Informatics and Telecommunications Engineering. Springer International Publishing; 2014. p. 144–7.

[32] Wang D, Park S, Fesenmaier DR. The Role of Smartphones in Mediating the Touristic Experience. J Travel Res. 2011;51(4):371–87. doi: 10.1177/0047287511426341

[33] Tussyadiah IP, Fesenmaier DR. Mediating tourist experiences: Access to places via shared videos. Ann Tour Res. 2009;36(1):24–40.

[34] Nielsen J. Usability Engineering. Morgan Kaufmann, San Francisco. 1993.

[35] Moorthy AK, Bovik AC. Blind image quality assessment: From natural scene statistics to perceptual quality. IEEE Trans Image Process. 2011;20(12):3350–64. 21521667 10.1109/TIP.2011.2147325

[36] Wu C, Yin S, Qi W, Wang X, Tang Z, Duan N. Visual chatgpt: Talking, drawing and editing with visual foundation models. arXiv preprint arXiv:2303.04671. 2023.

[37] Lu Y, Xue Z, Xia G-S, Zhang L. A survey on vision-based UAV navigation. Geo-spatial Inform Sci. 2018;21(1):21–32. doi: 10.1080/10095020.2017.1420509

[38] Stöcker C, Bennett R, Nex F, Gerke M, Zevenbergen J. Review of the Current State of UAV Regulations. Remote Sens. 2017;9(5):459. doi: 10.3390/rs9050459

[39] Finn RL, Wright D, Friedewald M. Seven Types of Privacy. European Data Protection: Coming of Age. Springer Netherlands; 2012. p. 3–32.

[40] Maza I, Caballero F, Capitán J, Martínez-de-Dios JR, Ollero A. Experimental Results in Multi-UAV Coordination for Disaster Management and Civil Security Applications. J Intell Robot Syst. 2010;61(1–4):563–85. doi: 10.1007/s10846-010-9497-5

